# How does obesity lead to insulin resistance?

**DOI:** 10.7554/eLife.33298

**Published:** 2017-12-14

**Authors:** Chan Hee J Choi, Paul Cohen

**Affiliations:** 1 Laboratory of Molecular MetabolismThe Rockefeller UniversityNew YorkUnited States

**Keywords:** adipocyte, insulin resistance, epigenetic, obesity, type 2 diabetes, glucose, Mouse

## Abstract

Experiments on mice show that an enzyme called DNA methyltransferase 3a is involved in insulin resistance via an epigenetic mechanism.

**Related research article** You D, Nilsson E, Tenen DE, Lyubetskaya A, Lo J, Jiang R, Deng J, Dawes BA, Vaag A, Ling C, Rosen ED, Kang S. 2017. Dnmt3a is an epigenetic mediator of adipose insulin resistance. *eLife* **6**:e30766. doi: 10.7554/eLife.30766

Insulin is a hormone produced by the pancreas that regulates the concentration of glucose in the blood (Saltiel and Kahn, 2001; Taniguchi et al., 2006). Following a meal, insulin secretion increases glucose uptake in muscle and fat, and reduces the production of glucose by the liver, enabling blood glucose levels to be maintained within a precise range. However, in cases of chronic overnutrition, the body can become less responsive to insulin, leading to a state known as insulin resistance. Initially, the pancreas responds by secreting more insulin, but when this can no longer compensate for the impaired insulin response of the liver and other organs, type 2 diabetes develops (Prentki and Nolan, 2006).

Insulin resistance serves as a key link between obesity and type 2 diabetes (Kahn and Flier, 2000), and understanding how obesity causes insulin resistance will improve our knowledge of type 2 diabetes and our ability to treat obesity-related complications. It is likely that the development of obesity-induced insulin resistance involves a complex interplay of genetic and environmental factors (Samuel and Shulman, 2012).

One mechanism by which cells can integrate signals from the environment is through epigenetic changes: these are changes that modify DNA or the proteins that organize the DNA within cells without changing the underlying DNA sequence (Jaenisch and Bird, 2003). DNA methylation, for example, is an epigenetic change that involves adding a methyl group to a cytosine base that is upstream of guanosine. Islands of these CpG sequences are often found in the gene promoters near the start of genes, and the methylation of these bases usually reduces gene expression. Now, in eLife, Sona Kang, Evan Rosen and colleagues in the USA, Denmark and Sweden – including Dongjoo You of the University of California Berkeley as first author – report how a key epigenetic regulator called *Dnmt3a* contributes to obesity-related insulin resistance (You et al., 2017).

The DNA methyltransferase (Dnmt) family of enzymes catalyze the methylation of DNA (Denis et al., 2011). You et al. noticed that fat cells taken from obese mice contained high levels of two DNA methyltransferases, and experiments on fat cells grown in the laboratory suggested that one of these enzymes – *Dnmt3a* – had a role in the development of insulin resistance. When the researchers knocked down or inhibited *Dnmt3a*, the fat cells were able to take up more glucose in response to insulin, even when they had been treated with substances that induce insulin resistance. Similar results were observed in genetically engineered mice that lacked *Dnmt3a* in their fat cells. When placed on a high fat diet, the knockout mice gained the same amount of weight as wild-type mice, but they demonstrated improved glucose and insulin tolerance, and their fat cells responded more strongly to insulin.

You et al. then performed a screen to check if blocking or overexpressing *Dnmt3a* changed the level of transcription of various genes. They found that a gene called *Fgf21*, which encodes a secreted protein that helps fat cells to absorb glucose (Kharitonenkov et al., 2005), was transcribed less when *Dnmt3a* was overexpressed. *Fgf21* is predominantly secreted by the liver, but fat cells are also known to express *Fgf21*. Based on their findings, You et al. suggest that *Dnmt3a* can induce insulin resistance in fat cells by methylating the CpG islands in the *Fgf21* promoter region. This reduces the expression of *Fgf21* genes, which in turn makes fat cells more resistant to insulin. Similar mechanisms might also be at play in human fat cells: people with diabetes had higher methylation levels near the *FGF21* gene compared to people without diabetes, and the degree of methylation was also inversely correlated with the levels of *FGF21* mRNA.

While insulin resistance may play a fundamental role in the development of type 2 diabetes in obese individuals, currently available anti-diabetic agents that target insulin resistance are limited to a single class of drugs called thiazolidinediones, which are no longer widely used due to potential side effects (Yki-Järvinen, 2004). DNA methylation has been an attractive therapeutic target in other clinical contexts, such as cancer. Therefore, targeting *Dnmt3a* or *Fgf21* could provide new treatment opportunities for obesity-related complications such as insulin resistance.

You et al.’s study raises several key questions that could be addressed in future studies. First, the mechanism underlying the upregulation of *Dnmt3a* in obesity is not clear. Second, the repression of *Fgf21* appears to explain only some of the downstream actions of *Dnmt3a*. Further research may be able to identify additional genes and pathways that are regulated by *Dnmt3a*, which could help with the development of alternative treatments for insulin resistance.
